# Health service quality scale: Brazilian Portuguese translation, reliability and validity

**DOI:** 10.1186/1472-6963-13-24

**Published:** 2013-01-17

**Authors:** Luiz Roberto Martins Rocha, Daniela Francescato Veiga, Paulo Rocha e Oliveira, Elaine Horibe Song, Lydia Masako Ferreira

**Affiliations:** 1 Plastic Surgery Post-graduation Program, Universidade Federal de São Paulo, Rua Napoleão de Barros, 715/4º andar, 04024-900, São Paulo, São Paulo, Brazil; 2 Division of Plastic Surgery, Department of Surgery, Universidade do Vale do Sapucaí, Avenida Prefeito Tuany Toledo, 470, 37550-000, Pouso Alegre, Minas Gerais, Brazil; 3 IESE Business School, Avinguda de Pearson, 21, 08034, Barcelona, Spain; 4 AllCells LLC, 5858 Horton Street, Emeryville, CA, 94608, USA

**Keywords:** Patient satisfaction, Psychometrics, Quality of health care, Scales

## Abstract

**Background:**

The Health Service Quality Scale is a multidimensional hierarchical scale that is based on interdisciplinary approach. This instrument was specifically created for measuring health service quality based on marketing and health care concepts. The aim of this study was to translate and culturally adapt the Health Service Quality Scale into Brazilian Portuguese and to assess the validity and reliability of the Brazilian Portuguese version of the instrument.

**Methods:**

We conducted a cross-sectional, observational study, with public health system patients in a Brazilian university hospital. Validity was assessed using Pearson’s correlation coefficient to measure the strength of the association between the Brazilian Portuguese version of the instrument and the SERVQUAL scale. Internal consistency was evaluated using Cronbach’s alpha coefficient; the intraclass (ICC) and Pearson’s correlation coefficients were used for test-retest reliability.

**Results:**

One hundred and sixteen consecutive postoperative patients completed the questionnaire. Pearson’s correlation coefficient for validity was 0.20. Cronbach's alpha for the first and second administrations of the final version of the instrument were 0.982 and 0.986, respectively. For test-retest reliability, Pearson’s correlation coefficient was 0.89 and ICC was 0.90.

**Conclusions:**

The culturally adapted, Brazilian Portuguese version of the Health Service Quality Scale is a valid and reliable instrument to measure health service quality.

## Background

The attainment of quality in products and services has become a general concern since the 1980s [1]. Especially in the health care sector, service quality is difficult to measure and often confounded with productivity [2]. It is important to understand that the quality of health services should be evaluated not in general terms, but rather in relation to specific dimensions, such as structure, process and outcome. These are fundamental approaches to quality assessment [3,4]. In this context, patient satisfaction and patient perception of quality of care are key concepts. Patient satisfaction may be defined as a personal evaluation of health services that cannot be assessed by direct observation. The patient’s opinion may be considered a subjective and multidimensional indicator of health service quality [5].

At present, clinicians and researchers evaluate the quality of health care delivered not only by monitoring treatment outcomes, such as complication rates and length of hospital stay, but also the patient’s perception of the treatment received [6,7]. Quality management in the service sector has been the subject of numerous studies on health care [8,9]. Patient satisfaction may be assessed with the use of instruments such as SERVQUAL [10] and the Health Service Quality Scale [11].

SERVQUAL is a multiple-item scale for assessing customers’ perception of service quality; it is a well-known instrument used in several areas of the service sector. The scale was developed considering that service quality is the difference between customers’ expectations for the service to be provided and their perceptions of the service received [10]. In addition, the SERVQUAL scale, a generic measure of service quality [10], was given to all participants (n =86) following the second administration of the final version of instrument. We used the culturally-adapted Brazilian-Portuguese version of the instrument, which was authorized by the original publishers and can be found in [12].

The Health Service Quality Scale is a multidimensional hierarchical scale based on interdisciplinary approach. This instrument was specifically created for measuring health service quality based on marketing and health care concepts; its primary dimensions are interpersonal quality, technical quality, environment quality, and administrative quality [11].

The purpose of this study was to translate and culturally adapt the Health Service Quality Scale into Brazilian Portuguese, and to assess the validity and reliability of the Brazilian Portuguese version of the instrument. The cultural adaptation process entails not only translating, but also adapting the scale for use in a cultural setting different from the one where the original study was conducted, thus reducing the possibility that the survey respondents will misinterpret the intended meaning of each of the instrument's items.

## Methods

The study was approved by the Research Ethics Committee of Unifesp - Federal University of São Paulo - under protocol number 2170/08 and performed in accordance with the ethical standards of the 1964 Declaration of Helsinki and its subsequent revisions. The process of translation into Portuguese was authorized by the authors of the original scale. Written informed consent was obtained from all patients prior to their inclusion in the study, and anonymity was assured. This was a cross-sectional study conducted with 116 consecutive patients selected at the Plastic Surgery Service of the Samuel Libânio University Hospital, which is part of the Brazilian public health system and provides health care services to the population of Southeastern Brazil.

The inclusion criteria comprised patients aged ≥18 years who had undergone inpatient surgery, were attending the Plastic Surgery Service for follow-up visits during the immediate postoperative period, and were registered in the hospital management system. Patients with history of psychiatric hospitalization or who were illiterate were excluded from the study.

### Instrument

The Health Service Quality Scale is a 73-item measure scored on a 7-point Likert-type scale (1 = completely disagree, 4 = neither agree nor disagree, and 7 = completely agree). This is a multidimensional hierarchical instrument for measuring health service quality that has the ability to predict important service outcomes, namely, patient satisfaction and behavioral intentions [11] (see Additional file 1: Appendix 1).

### Translation process

The translation process was performed according to established guidelines [13,14]. The original version of the Health Service Quality Scale [11] was initially translated into Brazilian Portuguese by two native Brazilian independent translators with previous experience in scientific translation, who were aware of the objective of the study. It was emphasized that the translation should be more conceptual than literal.

The two translations were compared by a multidisciplinary committee composed of four professionals: (1) a plastic surgeon, full Professor, doctoral advisor, with 28 years of experience in teaching, and MBA in management (LMF); (2) a Professor in Marketing in Europe, PhD in Management Science, with 10 years of experience in teaching in the US (PRO); (3) a plastic surgeon, PhD in Plastic Surgery, who also holds an MBA from a US institution, currently an Associate Senior Consultant for a health organization in the US (EHS); and (4) a dental surgeon, who holds Master’s degree in Dentistry, and was a doctoral student in Plastic Surgery Post-graduation Program at the time the study was conducted (LRMR).

The first consensual version of the translated instrument was back-translated by two other independent translators, who were not aware of the original questionnaire in English and objectives of the study. The two translators, one North-American and one British with 20 and 31 years of experience in translation, respectively, were members of the Brazilian Association of Translators (ABRATES). The multidisciplinary committee compared the original version with the translated and back-translated versions of the instrument, evaluated the differences between versions resulting from the translation process, and approved a consensual “Brazilian Portuguese version 2” of the instrument.

### Cultural adaptation

The Brazilian Portuguese version 2 of the Health Service Quality Scale was initially tested on a group of patients (*n* = 10) from the Plastic Surgery Service of the Samuel Libânio University Hospital. Undergraduate Human Resource Management and Technology students (collaborators) were responsible for providing the initial instructions to the patients, asking them to sign the informed consent form and monitoring the completion of the general information section of the questionnaire. Since this was a self-administered instrument, the collaborators remained in the room until the 73 items of the scale were completed by the patients.

At this stage, every difficulty in understanding the questions, doubts, and eventual suggestions of the participants regarding the questionnaire were recorded and later evaluated by the multidisciplinary committee. Eight issues were raised by the participants on items 3, 23, 33, 43, 50, 66, and 71. These items were subsequently modified using more appropriate wording. The revised questionnaire was given to a second group of patients (*n* = 20), who fully understood the content of the instrument.

The final Brazilian Portuguese version of the Health Service Quality Scale was then given to another group of 86 patients on two occasions four weeks apart. In addition, the culturally adapted Brazilian Portuguese version of the SERVQUAL scale [12], which is a generic measure of service quality [10], was given to all participants (*n* =86) following the second administration of the final version of instrument.

Upon conclusion of translation, cultural adaptation, and validity and reliability analyses, the Brazilian Portuguese version of the instrument, named the Health Service Quality Scale/Escola Paulista de Medicina (HSQS/EPM), was created (see Additional file 2: Appendix 2).

### Statistical analysis

Given that lack of a universally accepted method for determining sample sizes in validation studies, we relied on Bonett and Wright's [15] two-stage approximation to the sample size requirement for estimating Pearson correlations, as this was the statistic of interest in our study. In light of the high correlations between various SERVQUAL and the Health Service Quality scale observed in Dagger *et al.*'s original study [11] we also expected a high correlation coefficient in our study. Given an expected coefficient of 0.8 and assuming an interval width of .2 at a 95% confidence level, the required sample size is 56. Our convenience sample of 86 participants comfortably exceeds this requirement.

Validity was assessed using Pearson’s correlation coefficient to measure the strength of the association between the HSQS/EPM and the SERVQUAL scale. The standardized Cronbach's alpha coefficient was calculated for the two administrations of the HSQS/EPM to evaluate its internal consistency. Intraclass (ICC) and Pearson’s correlation coefficients were used for test-retest reliability; total scores obtained on the first and second administrations were used in the calculations.

The next step involved comparing the answers and the total scores between the HSQS/EPM and SERVQUAL scales through a paired test. As the normality assumption was not met, the Wilcoxon test was used in comparisons of scores. The normality assumption was evaluated using the Shapiro-Wilk test. The significance level, adopted was 5%.

The collected data were entered into an Excel spreadsheet and analyzed using R software version 2.7.1 [16].

## Results

The comparison between total scores of the HSQS/EPM and SERVQUAL scale is shown in Table 1. The total score for the SERVQUAL scale was significantly higher than that for the HSQS/EPM (*P* = 0.005). Pearson’s correlation coefficient between the two scales was 0.20 (*P* = 0.056). Cronbach's alpha for the first and second administrations of the HSQS/EPM were 0.982 and 0.986, respectively. For test-retest reliability, Pearson’s correlation coefficient was 0.89 (*P* < 0.001) and ICC was 0.90.

**Table 1:** **Comparison between total scores of the Health Service Quality Scale**/**Paulista School of Medicine** – **HSQS**/**EPM** (**first administration**) **and the SERVQUAL scale**

**Scales**	**N**	**Mean**	**SD**	**Min**.	**Q1**	**Median**	**Q3**	**Max**.	***P***-**value**
HSQS/EPM (1st administration)	86	74.6	4.6	60.0	72.0	75.5	78.0	86.4	0.005^1^
SEVQUAL scale	86	76.7	5.7	60.0	73.0	78.0	78.0	99.0	

N, number of participants; SD, standard deviation; Min., minimum; Q1, first quartile; Q3, third quartile Max., maximum; P, probability value.

^1^ Wilcoxon test.

Table 2 contains the Pearson Correlation Coefficient, p-value and Interclass Correlation Coefficient between the two applications of the Health Services Quality Scale for the seven scale items that had to be improved in terms of translation in the cultural adaptation (items 3, 23, 33, 43, 50, 66 and 71).

**Table 2:** **Pearson Correlation Coefficient, **“***P***”**value and Interclass Correlation Coefficient between the two applications of the Health Services Quality Scale**

**Question**	**Correlation**	***P*Value**	**ICC**
3	0,560	<0,001	0,75
23	0,761	<0,001	0,76
33	0,846	<0,001	0,85
43	0,896	<0,001	0,81
50	0,897	<0,001	0,88
66	0,698	<0,001	0,69
71	0,654	<0,001	0,66

## Discussion

There are 6,801 hospitals and 463,166 hospital beds in Brazil [17]. In 2007, Brazilian families spent US$ 77.3 billion (4.8% of the Brazilian gross domestic product) with health care goods and services, of which US$ 41.4 billion were spent with health care services [18]. Studies have indicated an urgent need for improvement in hospital performance in Brazil in both the private and public sectors [19,20]. This reflects the need for a professionalization process of hospital management, focusing on two main factors, namely, cost and quality of care. Professionalization of hospital management is one of the major challenges facing the Brazilian health policy. From a management point of view, health service quality is seen as a means of gaining competitive advantage and long-term profitability [21,22]. This can be achieved by improving the quality of services offered to patients with emphasis on patient satisfaction, which is now deemed a critical element in the day-to-day operation of health care institutions seeking high performance [23].

In the present study, validity of the HSQS/EPM was assessed by measuring the strength of the association between the final version of the translated instrument (a specific measure of health service quality) and the SERVQUAL scale (a generic measure of service quality). It is important to note that validity analysis does not determine whether an instrument is valid or not, but it shows the degree of validity of the instrument for specific purposes, and that it is always necessary to resort to empirical investigation to assess the validity of an instrument [24]. Therefore, it is inaccurate to state that an instrument “has been validated”, because validity is not a dichotomous variable, but refers to the internal indicators of an instrument and the strength with which these internal indicators correlate with external indicators.

In this context, a limitation of the present study is the lack of a cross-culturally validated measure, specific for measuring health service quality, with characteristics similar to those of the Health Service Quality Scale to be compared with the HSQS/EPM. We opted for the SERVQUAL scale, which is a widely used instrument for measuring health service quality, and one of the most traditional measures of service quality [10,25]. Limitations include the fact that the SERVQUAL scale is a generic instrument not specifically designed to measure health service quality, consisting of 22 items (while the HSQS/EPM has 73 items) comprising negatively worded statements that may confuse the respondents, in contrast to the HSQS/EPM that only has positively worded items.

On the other hand, the standardized Cronbach's alpha for the first and second administrations of the instrument were 0.982 and 0.986, respectively, showing that the HSQS/EPM has a high internal consistency and satisfy recommended minimum standards for reliability (*α* > 0.70), and indicating homogeneity of the items that comprise the scale [26-28]. For test-retest reliability, Pearson’s correlation coefficient (*r*) was 0.89 and ICC was 0.90, when considering the total scores obtained on the first and second administrations of the instrument. The value of *r* indicates that if the total score on the first administration is high then the total score on the second administration is also expected to be high, and vice-versa. The results were considered adequate, indicating that the HSQS/EPM is a reliable instrument [29].

The HSQS/EPM is part of the current trend that supports the use of instruments that have already been tested and validated in the field instead of developing new measures. The consolidation of the HSQS/EPM in the context of professionalization of the management of health services in Brazil may contribute to the development of scientific research on topics currently considered as priority by the international community, and to the management of health systems that add value to the patient and those that foster value-based competition on results, improving health care value for patients [30].

## Conclusions

The culturally adapted, Brazilian Portuguese version of the Health Service Quality Scale is a valid and reliable instrument to measure health service quality. This study has immediate implications for healthcare professions and managers, who can now confidently use the EQSS/EPM instrument described in this study in the measurement of health service quality in Brazil.

From the academic perspective, this study opens up a number of potentially interesting avenues for further research. First, it makes possible the comparison of perceived quality levels in health services in different Portuguese-speaking regions. A comparison across several regions of Brazil, a country of continental dimensions, or a comparison of rural and urban regions are of particular interest to the authors. Second, the instrument can also be applied to compare the quality of various units in the same hospital. Finally, there is the possibility of conducting international multi-cultural studies involving Portuguese and English speakers.

## Competing interests

The authors declare that they have no competing interests.

## Authors’ contributions

LRR participated in the design, carried out the study, interviewed the patients, prepared the dataset, analyzed and prepared all the results and drafted the manuscript. DFV participated in the design of the study and revised critically the manuscript. PRO, EHS and LMF conceived the study and designed it, helped to draft the manuscript and made important final modifications. All authors read and approved the final manuscript.

## Pre-publication history

The pre-publication history for this paper can be accessed here:

http://www.biomedcentral.com/1472-6963/13/24/prepub

## Supplementary Material

Additional file 1 **Items of the Health Service Quality Scale **[11]**.** Click here for file

Additional file 2 Health Service Quality Scale / Escola Paulista de Medicina (HSQS/EPM) in Brazilian Portuguese. Click here for file
