# Supplementary material for: Health service quality scale: Brazilian Portuguese translation, reliability and validity
Source: BMC Health Serv Res. 2013 Jan 17;13:24. doi: 10.1186/1472-6963-13-24 (PMC3579716; doi:10.1186/1472-6963-13-24)
Supplement: Additional file 1 — Items of the Health Service Quality Scale [11]. [file 1472-6963-13-24-S1.docx]

Additional file 1: Appendix 1

**Items of the Health Service Quality Scale** [11]

| **Health Service Quality Scale** |
| --- |
| Respondents rate the clinic’s performance on each scale item using a 7-point scale (1 = *strongly disagree*, 7 = *strongly agree*). |
| The items below are grouped by dimension for expositional convenience; they appear in random order on the survey. |
| **Perceived service quality** |
| The overall quality of the service provided by the clinic is excellent. |
| The quality of the service provided at the clinic is impressive. |
| The service provided by the clinic is of a high standard. |
| I believe the clinic offers service that is superior in every way. |
| **Service satisfaction** |
| My feelings towards the clinic are very positive. |
| I feel good about coming to this clinic for my treatment. |
| Overall I am satisfied with the clinic and the service it provides. |
| I feel satisfied that the results of my treatment are the best that can be achieved. |
| The extent to which my treatment has produced the best possible outcome is satisfying. |
| **Behavioral intentions** |
| If I had to start treatment again I would want to come to this clinic. |
| I would highly recommend the clinic to other patients. |
| I have said positive things about the clinic to my family and friends. |
| I intend to continue having treatment, or any follow-up care I need, at this clinic. |
| I have no desire to change clinics. |
| I intend to follow the medical advice given to me at the clinic. |
| I am glad I have my treatment at this clinic rather than somewhere else. |
| **Interpersonal quality** |
| The interaction I have with the staff at the clinic is of a high standard. |
| The interaction I have with the staff at the clinic is excellent. |
| I feel good about the interaction I have with the staff at the clinic. |
| **Technical quality** |
| The quality of the care I receive at the clinic is excellent. |
| The care provided by the clinic is of a high standard. |
| I am impressed by the care provided at the clinic. |
| **Environment quality** |
| I believe the physical environment at the clinic is excellent. |
| I am impressed with the quality of the clinic’s physical environment. |
| The physical environment at the clinic is of a high standard. |
| **Administrative quality** |
| The administration system at the clinic is excellent. |
| The administration at the clinic is of a high standard. |
| I have confidence in the clinic’s administration system. |
| **Interaction** |
| The staff at the clinic always listen to what I have to say. |
| The clinic’s staff treat me as an individual and not just a number. |
| I feel the staff at the clinic understand my needs. |
| The staff at the clinic are concerned about my well-being. |
| I always get personalized attention from the staff at the clinic. |
| I find it easy to discuss things with the staff at the clinic. |
| The staff at the clinic explain things in a way that I can understand. |
| The staff at the clinic are willing to answer my questions. |
| I believe the staff at the clinic care about me. |
| **Relationship** |
| The staff and I sometimes kid around, laugh, or joke with each other like close friends. |
| The staff and I talk about the things that are happening in our lives, and not just about my medical condition. |
| I have built a close relationship with some of the staff at the clinic. |
| **Outcome** |
| I feel hopeful as a result of having treatment at the clinic. |
| Coming to the clinic has increased my chances of improving my health. |
| I believe my future health will improve as a result of attending the clinic. |
| I believe having treatment at the clinic has been worthwhile. |
| I leave the clinic feeling encouraged about my treatment. |
| I believe the results of my treatment will be the best they can be. |
| **Expertise** |
| You can rely on the staff at the clinic to be well trained and qualified. |
| The staff at the clinic carry out their tasks competently. |
| I believe the staff at the clinic are highly skilled at their jobs. |
| I feel good about the quality of the care given to me at the clinic. |
| **Atmosphere** |
| The atmosphere at the clinic is pleasing. |
| I like the “feel” of the atmosphere at the clinic. |
| The clinic has an appealing atmosphere. |
| The temperature at the clinic is pleasant. |
| The clinic smells pleasant. |
| **Tangibles** |
| The furniture at the clinic is comfortable. |
| I like the layout of the clinic. |
| The clinic looks attractive. |
| I like the interior decoration (e.g., style of furniture) at the clinic. |
| The color scheme at the clinic is attractive. |
| The lighting at the clinic is appropriate for this setting. |
| The design of the clinic is patient friendly. |
| **Timeliness** |
| The clinic keeps waiting time to a minimum. |
| Generally, appointments at the clinic run on time. |
| **Operation** |
| The clinic’s records and documentation are error free (e.g., billing). |
| The clinic works well with other service providers (e.g., pathology). |
| I believe the clinic is well-managed. |
| The registration procedures at the clinic are efficient. |
| The discharge procedures at the clinic are efficient. |
| The clinic’s opening hours meet my needs. |
| **Support** |
| The clinic frequently runs support groups and programs for patients. |
| The clinic provides patients with an excellent range of support services. |
| The clinic provides patients with services beyond medical treatment. |
