# Supplementary material for: Health service quality scale: Brazilian Portuguese translation, reliability and validity
Source: BMC Health Serv Res. 2013 Jan 17;13:24. doi: 10.1186/1472-6963-13-24 (PMC3579716; doi:10.1186/1472-6963-13-24)
Supplement: Additional file 2 — Health Service Quality Scale / Escola Paulista de Medicina (HSQS/EPM) in Brazilian Portuguese. [file 1472-6963-13-24-S2.docx]

Additional file 2:Appendix 2

**Health Service Quality Scale / Escola Paulista de Medicina (HSQS/EPM) in Brazilian Portuguese**

| **Escala da Qualidade de Serviços de Saúde / Escola Paulista de Medicina – EQSS/EPM.** | | | | | | | | | | | |
| --- | --- | --- | --- | --- | --- | --- | --- | --- | --- | --- | --- |
| *Nº: ____________* | | | | | | | | | *DATA: ____/____/____* | | |
| a) Idade: ______ anos | | | | | | | | | | | |
| b) Gênero: | | □Masculino | | | □Feminino | | | | | | |
| c) Escolaridade: | | | | | | | | | | | |
| □ Ensino fundamental (1º grau) incompleto | | | | | | | | | | | |
| □ Ensino fundamental (1º grau) completo | | | | | | | | | | | |
| □ Ensino médio (2º grau) incompleto | | | | | | | | | | | |
| □ Ensino médio (2º grau) completo | | | | | | | | | | | |
| □ Superior completo (universitário) | | | | | | | | | | | |
| □ Pós Graduação | | | | | | | | | | | |
| d) Já esteve internado(a) para tratamento psiquiátrico? | | | | | | | | | | | |
| □SIM □NÃO | | | | | | | | | | | |
| e) Qual cirurgia você fez? ________________________________ | | | | | | | | | | | |
| **INSTRUÇÕES** | | | | | | | | | | | |
| Nós precisamos saber qual a sua opinião sobre os vários serviços prestados pela Cirurgia Plástica. | | | | | | | | | | | |
| Não existe resposta certa ou errada. | | | | | | | | | | | |
| O importante é a sua opinião. | | | | | | | | | | | |
| Seu nome não vai aparecer. | | | | | | | | | | | |
| Para cada uma das frases escolha um número, conforme a legenda abaixo: | | | | | | | | | | | |
| **Discordo totalmente** | | |  |  | | **Indiferente**  **(Neutro)** |  | | |  | **Concordo totalmente** |
| 1 | | | 2 | 3 | | 4 | 5 | | | 6 | 7 |
|  | A qualidade geral do serviço oferecido pelo Serviço de Cirurgia Plástica é excelente. | | | | | | | 1 2 3 4 5 6 7 | | | |
|  | A qualidade do serviço oferecido no Serviço de Cirurgia Plástica é impressionante. | | | | | | | 1 2 3 4 5 6 7 | | | |
|  | O serviço oferecido pelo Serviço de Cirurgia Plástica é de alto padrão. | | | | | | | 1 2 3 4 5 6 7 | | | |
|  | Acho que o Serviço de Cirurgia Plástica oferece serviço de qualidade superior em todos os seus níveis. | | | | | | | 1 2 3 4 5 6 7 | | | |
|  | Minha impressão sobre o Serviço de Cirurgia Plástica é muito positiva. | | | | | | | 1 2 3 4 5 6 7 | | | |
|  | Eu me sinto bem em vir ao Serviço de Cirurgia Plástica para meu tratamento. | | | | | | | 1 2 3 4 5 6 7 | | | |
|  | De forma geral, estou satisfeito com o Serviço de Cirurgia Plástica e com o tratamento que ele oferece. | | | | | | | 1 2 3 4 5 6 7 | | | |
|  | Acredito que os resultados do meu tratamento são os melhores que podem ser alcançados. | | | | | | | 1 2 3 4 5 6 7 | | | |
|  | Estou satisfeito que meu tratamento tenha produzido o melhor resultado possível. | | | | | | | 1 2 3 4 5 6 7 | | | |
|  | Caso precisasse recomeçar o tratamento, eu escolheria este Serviço de Cirurgia Plástica. | | | | | | | 1 2 3 4 5 6 7 | | | |
|  | Recomendaria este Serviço de Cirurgia Plástica sem qualquer restrição a outros pacientes. | | | | | | | 1 2 3 4 5 6 7 | | | |
|  | Tenho feito comentários positivos sobre o Serviço de Cirurgia Plástica para a minha família e amigos. | | | | | | | 1 2 3 4 5 6 7 | | | |
|  | Pretendo continuar meu tratamento, ou receber o acompanhamento de que precisar, neste Serviço de Cirurgia Plástica. | | | | | | | 1 2 3 4 5 6 7 | | | |
|  | Não desejo mudar de Serviço de Cirurgia Plástica. | | | | | | | 1 2 3 4 5 6 7 | | | |
|  | Pretendo seguir as orientações médicas que me foram dadas no Serviço de Cirurgia Plástica. | | | | | | | 1 2 3 4 5 6 7 | | | |
|  | Eu estou feliz por fazer o meu tratamento neste Serviço de Cirurgia Plástica e não em outro local. | | | | | | | 1 2 3 4 5 6 7 | | | |
|  | O entrosamento que eu tenho com a equipe do Serviço de Cirurgia Plástica é de alto padrão. | | | | | | | 1 2 3 4 5 6 7 | | | |
|  | O entrosamento que eu tenho com a equipe do Serviço de Cirurgia Plástica é excelente. | | | | | | | 1 2 3 4 5 6 7 | | | |
|  | Estou satisfeito com o entrosamento que tenho com a equipe do Serviço de Cirurgia Plástica. | | | | | | | 1 2 3 4 5 6 7 | | | |
|  | A qualidade do tratamento que recebo no Serviço de Cirurgia Plástica é excelente. | | | | | | | 1 2 3 4 5 6 7 | | | |
|  | A assistência prestada pelo Serviço de Cirurgia Plástica é de alto padrão. | | | | | | | 1 2 3 4 5 6 7 | | | |
|  | Estou bem impressionado com a assistência oferecida pelo Serviço de Cirurgia Plástica. | | | | | | | 1 2 3 4 5 6 7 | | | |
|  | Acho que o ambiente físico no Hospital Samuel Libânio / Serviço de Cirurgia Plástica é excelente. | | | | | | | 1 2 3 4 5 6 7 | | | |
|  | Estou impressionado com a qualidade do ambiente físico do Hospital Samuel Libânio / Serviço de Cirurgia Plástica. | | | | | | | 1 2 3 4 5 6 7 | | | |
|  | O ambiente físico do Hospital Samuel Libânio / Serviço de Cirurgia Plástica é de alto padrão. | | | | | | | 1 2 3 4 5 6 7 | | | |
|  | O sistema de administração do Serviço de Cirurgia Plástica é excelente. | | | | | | | 1 2 3 4 5 6 7 | | | |
|  | A administração do Serviço de Cirurgia Plástica é de alto padrão. | | | | | | | 1 2 3 4 5 6 7 | | | |
|  | Tenho confiança no sistema de administração do Serviço de Cirurgia Plástica. | | | | | | | 1 2 3 4 5 6 7 | | | |
|  | A equipe do Serviço de Cirurgia Plástica sempre ouve o que eu tenho a dizer. | | | | | | | 1 2 3 4 5 6 7 | | | |
|  | A equipe do Serviço de Cirurgia Plástica me trata como uma pessoa e não apenas como um número. | | | | | | | 1 2 3 4 5 6 7 | | | |
|  | Sinto que a equipe do Serviço de Cirurgia Plástica compreende as minhas necessidades. | | | | | | | 1 2 3 4 5 6 7 | | | |
|  | A equipe do Serviço de Cirurgia Plástica se preocupa com o meu bem estar. | | | | | | | 1 2 3 4 5 6 7 | | | |
|  | Sempre recebo atenção personalizada por parte da equipe do Serviço de Cirurgia Plástica. | | | | | | | 1 2 3 4 5 6 7 | | | |
|  | Acho fácil conversar as coisas com a equipe do Serviço de Cirurgia Plástica. | | | | | | | 1 2 3 4 5 6 7 | | | |
|  | A equipe do Serviço de Cirurgia Plástica explica as coisas de forma que eu consigo entender. | | | | | | | 1 2 3 4 5 6 7 | | | |
|  | A equipe do Serviço de Cirurgia Plástica demonstra disposição para responder às minhas perguntas. | | | | | | | 1 2 3 4 5 6 7 | | | |
|  | Acredito que a equipe do Serviço de Cirurgia Plástica se preocupa comigo. | | | | | | | 1 2 3 4 5 6 7 | | | |
|  | Às vezes, a equipe e eu fazemos brincadeiras, rimos, ou falamos de coisas do dia-a-dia como bons amigos. | | | | | | | 1 2 3 4 5 6 7 | | | |
|  | A equipe do Serviço de Cirurgia Plástica e eu conversamos sobre outras coisas que estão acontecendo em nossas vidas, e não apenas sobre a minha condição de saúde. | | | | | | | 1 2 3 4 5 6 7 | | | |
|  | Desenvolvi um bom relacionamento com alguns funcionários do Serviço de Cirurgia Plástica. | | | | | | | 1 2 3 4 5 6 7 | | | |
|  | Eu me sinto esperançoso por ter feito o tratamento no Serviço de Cirurgia Plástica. | | | | | | | 1 2 3 4 5 6 7 | | | |
|  | Ter frequentado o Serviço de Cirurgia Plástica aumentou as minhas chances de melhorar a minha saúde. | | | | | | | 1 2 3 4 5 6 7 | | | |
|  | Acredito que minha saúde futura irá melhorar em razão de frequentar o Serviço de Cirurgia Plástica. | | | | | | | 1 2 3 4 5 6 7 | | | |
|  | Acredito que valeu a pena fazer o tratamento no Serviço de Cirurgia Plástica. | | | | | | | 1 2 3 4 5 6 7 | | | |
|  | Saio do Serviço de Cirurgia Plástica sentindo-me encorajado sobre o meu tratamento. | | | | | | | 1 2 3 4 5 6 7 | | | |
|  | Acredito que os resultados do meu tratamento serão os melhores possíveis. | | | | | | | 1 2 3 4 5 6 7 | | | |
|  | Pode-se confiar que a equipe do Serviço de Cirurgia Plástica é bem treinada e qualificada. | | | | | | | 1 2 3 4 5 6 7 | | | |
|  | A equipe do Serviço de Cirurgia Plástica executa suas tarefas com competência. | | | | | | | 1 2 3 4 5 6 7 | | | |
|  | Acredito que a equipe do Serviço de Cirurgia Plástica é altamente capacitada para seus trabalhos. | | | | | | | 1 2 3 4 5 6 7 | | | |
|  | Eu me sinto satisfeito em relação à qualidade dos cuidados dispensados a mim no Serviço de Cirurgia Plástica. | | | | | | | 1 2 3 4 5 6 7 | | | |
|  | O ambiente do Hospital Samuel Libânio / Serviço de Cirurgia Plástica é agradável. | | | | | | | 1 2 3 4 5 6 7 | | | |
|  | Eu gosto da “sensação” do ambiente no Hospital Samuel Libânio / Serviço de Cirurgia Plástica. | | | | | | | 1 2 3 4 5 6 7 | | | |
|  | O Hospital Samuel Libânio / Serviço de Cirurgia Plástica tem um ambiente atraente. | | | | | | | 1 2 3 4 5 6 7 | | | |
|  | A temperatura no interior do Hospital Samuel Libânio / Serviço de Cirurgia Plástica é agradável. | | | | | | | 1 2 3 4 5 6 7 | | | |
|  | O Hospital Samuel Libânio / Serviço de Cirurgia Plástica tem um cheiro agradável. | | | | | | | 1 2 3 4 5 6 7 | | | |
|  | Os móveis do Hospital Samuel Libânio / Serviço de Cirurgia Plástica são confortáveis. | | | | | | | 1 2 3 4 5 6 7 | | | |
|  | Eu gosto do estilo visual do Hospital Samuel Libânio / Serviço de Cirurgia Plástica. | | | | | | | 1 2 3 4 5 6 7 | | | |
|  | O Hospital Samuel Libânio / Serviço de Cirurgia Plástica parece ser atraente. | | | | | | | 1 2 3 4 5 6 7 | | | |
|  | Gosto da decoração interior (ex.: estilo dos móveis) do Hospital Samuel Libânio / Serviço de Cirurgia Plástica. | | | | | | | 1 2 3 4 5 6 7 | | | |
|  | As cores utilizadas nas dependências do Hospital Samuel Libânio / Serviço de Cirurgia Plástica são atraentes. | | | | | | | 1 2 3 4 5 6 7 | | | |
|  | A iluminação no Hospital Samuel Libânio / Serviço de Cirurgia Plástica é adequada para este tipo de ambiente. | | | | | | | 1 2 3 4 5 6 7 | | | |
|  | O projeto do espaço físico do Hospital Samuel Libânio / Serviço de Cirurgia Plástica é acolhedor para o paciente. | | | | | | | 1 2 3 4 5 6 7 | | | |
|  | O tempo de espera no Serviço de Cirurgia Plástica é o mínimo possível. | | | | | | | 1 2 3 4 5 6 7 | | | |
|  | Em geral, as consultas no Serviço de Cirurgia Plástica são atendidas no horário marcado. | | | | | | | 1 2 3 4 5 6 7 | | | |
|  | Os registros e a documentação do Serviço de Cirurgia Plástica não apresentam erros (por exemplo, o sistema de tesouraria). | | | | | | | 1 2 3 4 5 6 7 | | | |
|  | O Serviço de Cirurgia Plástica trabalha bem com outros prestadores de serviço (por exemplo: patologia). | | | | | | | 1 2 3 4 5 6 7 | | | |
|  | Acredito que o Serviço de Cirurgia Plástica é bem administrado. | | | | | | | 1 2 3 4 5 6 7 | | | |
|  | Os procedimentos de registro de entrada no Serviço de Cirurgia Plástica são eficientes. | | | | | | | 1 2 3 4 5 6 7 | | | |
|  | Os procedimentos de saída (alta) do Serviço de Cirurgia Plástica são eficientes. | | | | | | | 1 2 3 4 5 6 7 | | | |
|  | Os horários de funcionamento do Serviço de Cirurgia Plástica atendem às minhas necessidades. | | | | | | | 1 2 3 4 5 6 7 | | | |
|  | O Serviço de Cirurgia Plástica frequentemente organiza grupos e programas de apoio para pacientes. | | | | | | | 1 2 3 4 5 6 7 | | | |
|  | O Serviço de Cirurgia Plástica disponibiliza uma excelente variedade de serviços de apoio aos pacientes. | | | | | | | 1 2 3 4 5 6 7 | | | |
|  | O Serviço de Cirurgia Plástica oferece aos pacientes serviços que vão além do tratamento médico. | | | | | | | 1 2 3 4 5 6 7 | | | |
